# The Challenges of Searching for Workforce Data About New Roles in NHS Mental Health Services: A Cross-Sectional Observation Study

**DOI:** 10.1177/00469580251399386

**Published:** 2025-12-01

**Authors:** Sally Sanger, Damian E. Hodgson, Emily Wood, Pauline A. Nelson, Jaqui Long

**Affiliations:** 1 The University of Sheffield, UK

**Keywords:** access to information, data quality, information sharing, information storage and retrieval, NHS mental health workforce, workforce data

## Abstract

High quality information is essential for good workforce planning and development, and it is therefore important to have accurate data about roles within services. NHS mental health services have been under great pressure for several years with insufficient staff, high turnover and overwhelming demand. To help address this a variety of new staff roles have been introduced by mental health Trusts, for example, Clinical Associates in Psychology, Mental Health, and Wellbeing Practitioners. This paper describes in detail the difficulties encountered in mapping where these new roles have been introduced in England and how the challenges were addressed. Data was obtained through 26 structured interviews and 5 questionnaires from appropriate mental health Trust representatives in a variety of different leadership roles (eg, clinical leads, human resources leads, workforce development leads). The data was then sense-checked in 3 stakeholder reflection groups with 18 participants including representatives of professional bodies, service providers and policy makers. The data indicated that (1) NHS mental health workforce information is unexpectedly difficult to obtain and (2) sometimes of poor quality, eg, inaccurate or incomplete. Challenges obtaining this data included: • Fragmented responsibility within trusts for data relating to new roles. • Inconsistent nomenclature. • Coding and retrieval issues with the Electronic Staff Record (ESR). The paper concludes that changes to NHS mental health workforce data on new roles are needed, including better access to and a higher quality of data. It offers recommendations for improvement, including role naming consistency and allocation of responsibility for an overview of new roles data.

**Highlights**

- Good workforce planning and development requires accurate information about existing staff roles and numbers.
- Many new roles have been introduced recently in English NHS mental health services.
- This study discusses the challenges encountered in practice in mapping these new mental health roles. Issues include: ● Fragmented responsibility within trusts for data relating to new roles, making it difficult to obtain ● Inconsistent / confusing use of terminology and names of roles, ● Coding and retrieval issues with the Electronic Staff Record (ESR).
- Many of the issues identified here have been in existence since at least 2008.
- The study highlights the need to address this with recommendations for NHS England, mental health services and researchers.

## Introduction

This paper discusses recent challenges encountered in collecting workforce data on new mental health (MH) roles from NHS MH Trusts in England (N = 54). It presents how the research team dealt with the challenges and offers recommendations for addressing them.

### Context

The research reported in this paper was part of a project conducted at The University of Sheffield, with the overall aim of understanding how NHS MH services can implement new roles and changes in skill mix in a way that maximises benefits for staff, service users, and organisations. This report draws on data from the project’s first 2 stages. The first stage aimed to generate a picture of which new roles had been introduced in each English NHS MH Trust. It sought to map where new roles were located and how many there were, drawing on semi-structured online interviews with appropriate leads in each Trust. It is the issues connected with mapping that are covered in this paper.

In the second stage, 3 reflection groups were organised to review the findings from the first stage. The data from these groups is also drawn on here as it provides additional insight into the challenge of generating quality data for research, policy and operational decision-making.

Workforce statistics are primarily drawn from the Electronic Staff Record (ESR) which is the NHS’s human resources and payroll record system used throughout the organisation. It was introduced between 2006 and 2008 and is continually developing, including with the introduction of codes for new roles. It can be used by staff (eg, to view payslips, find training courses) and managers (eg, for absence reporting, workforce reports).^1^ Staff can search for numbers of staff using report generation functions with appropriate filters.

### Research Questions

This paper seeks to address the following:

**RQ1**: What obstacles to securing quality data are encountered when mapping new roles provision within English NHS mental health Trust services?
**RQ2:** How might these obstacles be addressed?

### Literature Review

#### Mental Health Services

NHS staff shortages are well-known and are particularly acute in the MH sector where there are gaps in staff numbers, high turnover in some roles, and difficulties filling vacancies.^2-4^ At the same time demand for services has increased, in part due to the COVID-19 pandemic, especially among children, young people, and socially deprived groups.^2,3^ To help address workforce shortages and improve care, many new roles have been introduced to the MH workforce in England in recent years for example, Clinical Associates in Psychology (CAPs), Advanced Clinical Practitioners (ACPs), Mental Health Wellbeing Practitioners (MHWPs), Education Mental Health Practitioners (EMHPs), Peer Support Workers (PSWs), and Nursing Associates (NAs) amongst others. The new roles were introduced rather than recruiting existing roles (eg, psychologists, nurses) for a range of reasons. This includes difficulties with recruitment of existing roles, the wish to introduce new ways of working (eg, drawing on lived experience expertise), the need for cost-effectiveness and to embrace diversity by opening the roles to staff from a range of professional backgrounds.

There have been evaluations of the impact of some of these roles.^5-9^ For example, Slender and Taylor^5^ looked at the impact of the CAP role on acute MH wards during a 6-month period, finding that they improved access to psychological interventions including numbers of patient self-referrals. The study also assessed the role’s impact on the ward team with a focus group, finding that positive benefits included improvements in staff psychological knowledge and skills, and the confidence to use these. Jagielska-Hall and O’Driscoll’s^8^ analysis of a case series study of CAPs found a range of positive impacts including in relation to delivery of brief interventions, advice to other staff, service improvements, and contributing to population health strategies. A small study of the impact of MHWPs work with older adults^9^ found clinically significant improvements in service users.

Ellins et al,^6^ included evaluation of EMHPs in their evaluation of a Trailblazer MH programme, finding them to be popular but noting difficulties with retention and that they tended to focus on individual children rather than a ‘whole school’ approach. White et al,^7^ systematically reviewed the evidence on the effectiveness of one-to-one peer support. They noted incomplete reporting in many of the trials, but that they did suggest some psycho-social improvements for example, self-reported recovery and empowerment. However, the collective impact of the introduction of many new roles across MH services is not well understood yet.

#### Mental Health Service Data

Previous research on MH services data tended to focus on information about clinical outcomes and service provision, rather than workforce numbers. It has noted many problems, including gaps in patient experience and outcomes data,^4^ issues with quality^4^ for example, timeliness and comprehensiveness, outdated systems and duplicate data entry.^3^ In 2024 the King’s Fund stated that MH was often viewed as less important than physical health and that this attitude was reflected in data collection in terms of gaps in data and data completeness and quality.^3^

A recent rapid evidence review^10^ drawing on 300 experts examined the quality of data and evidence generally in MH. This identified multiple problems including that whilst data is often incomplete, the burden of data collection on staff is excessive. These problems made it difficult to know how the sector was performing, whether targets were being met, and how to plan and provide care. The review also specifically noted a need for ‘more systematic metrics on environment and workforce’.^10^ Other research has also noted problems with MH data generally: Davidson^11^ found that despite the large amount of data collected by NHS MH personnel the quality was poor with inconsistencies and inefficiencies. He notes that this affects the ability to judge service effectiveness and so to develop improvement plans. These inefficiencies also contributed to excess information requests from commissioning and regulatory bodies, adding to the pressure on staff.

A 2023 report from the National Audit Office^12^ (NAO) stated that improvements were being made in data and information, including the introduction of the Mental Health Services Data Set and publication of information on service activity and performance against new standards. However, they concluded that overall MH data remained a ‘red’ risk area with progress in developing MH datasets lagging behind other areas.

#### Mental Health Workforce Data

Addressing workforce development and workforce problems requires good data about what already exists to build appropriate plans and ensure effective services that are value for money.^13^ With staffing being the costliest element in the NHS, attempts to improve cost-effectiveness and quality are clearly dependent on workforce data.^14^ Fanneran et al,^15^ also noted the importance of evidence-based data on staffing to workforce plans. If accurate data cannot be found for a service, then modelling future improvements becomes very difficult and error prone.

Data on the NHS workforce overall has improved over the last 5 years, with increased use of digital platforms, more standardised data formats, improvements to the ESR, and integration of data sources. However, Davidson^11^ found problems with data quality specifically in relation to the mental health workforce. This included the reporting of staff numbers per discipline, lack of understanding of what staff do and duplicate data collection activities leading to lack of confidence in the data and the waste of resources.^11^

#### New Roles and Mental Health Workforce Data

There is very little written specifically about new roles and MH workforce data in England. Fanneran et al^15^ provides a short comment in his analysis of changes in the MH nursing workforce that there is no workforce national data for Advanced Clinical Practitioners in MH trusts. This is not applied to other roles, so it is a highly specific finding.

The only paper dealing with the topic at length found to date was Dickinson et al^16^ This discussed the data issues encountered in mapping 4 new MH roles introduced in 2004 to 2006. Dickinson et al., found in particular that there were problems regarding:

- The criteria used to define roles
- Clarity of terminology
- Lack of knowledge of where new staff members were located.^16^

Whilst there are striking similarities to the present paper, as will be shown, Dickinson et al.’s work reflects the situation 20 years ago, the roles studied are different and are only in community MH services. This report also covers data in inpatient services and provides a more up-to-date picture of the situation.

## Methods

### Ethical Approval

This research is in accordance with the World Medical Association Declaration of Helsinki. The research received institutional research ethics approval (ref 053435) on 06.07.23 and Health Research Authority approval (ref 23/HRA/3186) on 08.08.23.

### Study Design

This cross-sectional, observation study has a sequential 2-phase design. Stage 1 consisted of semi-structured online interviews with purposively sampled leaders from all NHS MH trusts (N = 54) in England to map the provision of new roles, their names and numbers (Where time allowed, respondents were asked about the rationale for having a role, its impact and the experience of introducing new roles. This data is reported in a separate paper currently under review.). Interviews were preferred to questionnaires as more likely to achieve results and as they enabled follow-up questions to be asked by both parties to aid discussion and timely clarification.^17,18^ The interviews were held between October 2023 and January 2024. The study was confined to all English MH NHS trusts to achieve a homogeneous sample as NHS structures and data collection differ in the other nations. Inclusion criteria for the staff approached to provide the data were that they should be at a senior enough level to have access to the data and be allowed to provide it to us.

Stage 2 consisted of 3 stakeholder reflection groups held in January/February 2024 with a range of purposively selected participants from for example, policy-making, provider or professional bodies. Using this method enabled us to sense check with a range of experts the data obtained in Stage 1, particularly the research team’s interpretation of the data. The inclusion criteria were that they should be senior level stakeholders with an interest or expertise in new roles, chosen for maximum variation of leadership role.

The data were analysed using descriptive statistics and Template Analysis^19^ (a form of thematic analysis). Coding was carried out in NVivo v14 by a highly experienced qualitative researcher, with checking carried out by 2 other coders.

### Stage 1: Interviews

The team took a robust approach to obtaining data on staff employed in new roles in English MH Trusts. This combined rigour with persistence, pragmatism and ingenuity. The process of recruiting appropriate informants was in practice unexpectedly complicated and time-consuming as many leads needed to be followed for some Trusts. This is further discussed in the results section below.

The team began by developing a list of contact names from each MH Trust’s website. This focussed initially on individuals such as workforce leads, HR leads, or senior clinicians/managers. Fifty-one Trusts were then approached to obtain direct contact information for this person usually via the switchboard. This number excludes 3 of our partner Trusts where personal contacts were used from the start. Once contact details had been obtained, the person was invited to interview (see Appendix A in Supplemental Materials for documents used). The alternative of a questionnaire was also offered (see Appendix B in Supplemental Materials) but only sent if requested.

Personnel agreeing to an interview or questionnaire were sent an information sheet and completed an online consent form. Once consent was obtained, a pre-interview sheet with examples of roles to set the context was sent to interviewees and a date for interview arranged. The interview guide asked about specific roles that the research team’s preliminary research and project stakeholder engagement indicated were common or important, plus others that emerged as the study proceeded. Participants were also asked to tell us about any other new roles that had not come to the research team’s attention and were asked to provide specific numbers. The interview recruitment materials and interview guide are available in Supplemental Materials, Appendix A and the questionnaire is in Appendix B.

Interviews were conducted online using Microsoft Teams (mean length 45 min, range 24-70 min) during September 23 to February 24, by when data saturation had been achieved. Video and audio were captured, downloaded and converted to audio-only recordings. Qualitative notes on the interviews were written and data giving the names of new roles and numbers/estimates of prevalence within the trust were extracted and entered on a spreadsheet. Seven interviewees sent the research team additional names of roles and numbers or estimates after the interview, which were included in the data.

### Stage 2: Stakeholder Reflection Groups

The 3 groups included a range of stakeholders, for example, service providers, policy makers, educators, and representatives of professional groups. The 18 participants were obtained by purposive sampling, having been identified during research proposal preparation or during the mapping stage. They gave informed consent via a form on Qualtrics. The groups were held on MS Teams during January to February 2024 (mean length of 85 min, range 78-93 min). The preliminary findings were discussed, and participants’ views obtained. This included discussion of the availability and quality of workforce data. The recordings were transcribed and the parallel Teams online chat for the meeting was downloaded and incorporated into the transcript.

## Results

### Data Obtained on New Roles

The number of interviews held, and questionnaires sent and returned was as given in Table 1 at the end of this text. Data from Stage 1 was obtained from 31/51 Trusts (response rate 61%) and consisted of 26 interviews and 5 returned questionnaires. Respondent Trusts covered all regions of England to varying degrees, and the response rate did not appear to correspond with any identifiable factor for example, location, size, or Care Quality Commission rating. This suggests that although the data was not complete (which is a limitation on its reliability) there was an absence of bias in the sample obtained. There was no bias in the sample approached as it consisted of all relevant trusts. Ninety-eight ‘new’ roles were initially identified, reduced to 85 after data had been cleaned.

**Table 1.** Interviews and Questionnaires.

Interviews conducted	26
Questionnaires requested as possible alternative	20
Questionnaires completed and returned	5

Rich qualitative data was obtained from the interviews and focus groups. Please note that this is primarily reported in a separate paper currently under peer review. It is drawn on here only as it illuminates the dilemmas encountered by the researchers in obtaining numbers of roles per trust, which is the focus of this paper. These numbers were unexpectedly difficult to obtain and indicated several important challenges with MH workforce data which are discussed in the remainder of this article.

Table 2 indicates a sample of the data ultimately obtained. Cells in dark grey depict where an exact figure for a role was supplied (please note that figure may be any specific number including zero). Light grey signifies that a qualitative description (eg, ‘we have some of those’) was given but no precise figure was retrievable. White indicates that no data on the role could be provided (meaning the Trust either has no data on that role or they had data, but the person interviewed was unable to retrieve it). Table 2 shows the 26 most cited roles and is provided so that readers can see the fragmented nature of the data, not to illustrate exact numbers. Data for the remaining roles was much sparser.

**Table 2.** The Availability of Accurate Data on New Roles.

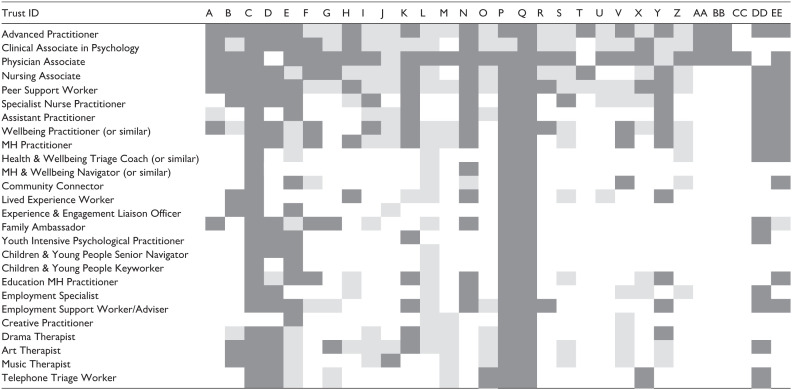

*Note.* Codes along the top of the table indicate the trust’s individual ID and the names of roles are given down the left hand side. Dark grey box – exact number provided; Light grey box – rough estimate provided for example, some, lots, a few; White box – no data provided.

### Challenges Experienced in Collecting Estimates of Role Prevalence in Trusts (Please note in this section quotations coded with a single letter and number are from the Stage 1 interviews, but those starting with SRG are from the Stage 2 Stakeholder Reflection Groups.)

#### Obtaining Initial and Subsequent Contacts

There was not always an obvious listing on the Trust website or inclusion of contact details for individuals who we decided to approach. Switchboard staff also were not usually allowed to give out direct phone numbers and did not always know the possible contact’s P.A. Occasionally they were not allowed to give out individual email addresses. Switchboards were sometimes uncertain where to direct people within a department for example, HR.

In many cases it emerged that the first individual invited was not the right person to interview. A range of strategies were then employed to reach someone able to obtain the data needed and willing to provide it. Sometimes the first contact could direct the team on, other times the researchers had to pursue new avenues. Different routes used to find the right person to provide the data included: via the Trust Board Secretary, R & D departments, professional colleagues of research team members, NHS England regional workforce leads, and a Communication Department. Numbers of contacts made per Trust at this stage ranged from 1 to 15, with only 11 Trusts receiving 5 or fewer contacts. The total number of contacts before either an interview was set up, the Trust declined, or time ran out are given in Table 3 and amounted to a total of 354 contacts with 210 individuals.

**Table 3.** Contact Information.

Contacts with trust personnel	233 Contacts with 155 individuals
Contacts with trust R & D department	97 Contacts with 41 individuals
Contacts with NHS England staff	13 Contacts with 4 individuals
Contacts with personal contacts	11 Contacts with 10 individuals
Total	354 Contacts with 210 individuals

The following presents an example using facts taken from a spreadsheet recording individual contacts with trusts. It is quoted below in full to illustrate the real-life complexities and time that could be involved in simply identifying the right person to invite from 1 trust. To summarise, it involved:

- Thirty separate actions (eg, emails sent, discussion held between Trust contacts),
- Communication with at least 10 people at the Trust

In this instance it took 3 months to find the right person and set up the interview. As well as Trust uncertainty about who could provide the data needed, reasons for the length of time here included technical delays (eg, ‘relay access denied’) and Trust staff absences or non-response. There was also one ‘false start’: a formal consent to interview by contact ‘AA’ that did not materialise in an interview despite multiple attempts to contact the individual.

Researcher 1 (R1) sent an invitation letter via the Board Secretary from the project email address asking for it to be forwarded to the Director of Nursing (DN) or her Personal Assistant (PA) 13.9.23. 14.9.23 DN replied to say not the best person for this and said she would forward to the Divisional Director (DD) for MH. R1 replied to thank them and ask for the name of DD and received an out of office reply until 20.9.23. 27.09.23 R1 asked DN again for the name of the DD or to forward a reminder to them. Bounced back saying relay access denied. Researcher 2 (R2) followed up from personal account 10.10.23 cc'ing DN's PA and the Trust Board email address. [The researcher’s personal account was used in case the system had mistakenly identified the New Roles account as spam.] DN replied to say that AA and BB are the best contacts. R2 invited AA and BB 10.10.23. AA completed a consent form. R2 followed them up for an interview slot 11.10.23. R2 chased 18.10.23 and 25.10.23. As a consent form had been completed, R2 followed up one last time 31.10.23.

Researcher 3 (R3) then contacted a personal contact at NHS England for assistance, 10.11.23 and chased them on 20.11.23. They suggested CC and DD. R2 invited them 29.11.23. R3 followed up 01.12.23 and R2 followed up 05.12.23. DD replied 05.12.23 to say that she had passed it to their Director of People (DP), who was discussing the Trust's possible involvement with its research department. DP completed the consent form on 12.12.23 and R2 set up an interview date. The interview took place on 18.12.2023.

#### Fragmented Responsibility for Data

Once a contact had been found, a full set of workforce data was not always accessible or understood by this one individual, as responsibility was often split between different leads.

One person might be confident about numbers for certain roles (eg, nursing), but only able to estimate other figures (eg, for psychological professions):

I can talk on behalf of our directorate but. . .I can’t speak for [the other directorates] (Interview D1. See also G1, M1, V1, X1).

Sometimes participants struggled to interpret job titles on NHS Trust systems:

I think it’s a real hodgepodge of different folks that are being put into that category [Wellbeing Practitioners] to the extent that I think as a single category I don’t really know what it means (SRG3-C)

This led to incomplete data and use of estimates.

#### Lack of Clarity in Terms Used by Respondents

There were inconsistencies around interpretation of the meaning of ‘new’. Trusts included a mixture of completely new roles, roles that were new to the Trust but not nationally new, and roles established in another service/clinical area but being used in new settings.

some are absolutely new roles like the responsible clinician, and a new way of thinking about how we support people for instance, but some such as mental health practitioner, isn’t necessarily a new role at all, it’s a new title. (SRG2-F)

A limitation of the study is that whilst interview participants were asked to specify what they meant by ‘new’ (see Interview Guide, Appendix A in the Supplemental Materials) this was not done with the questionnaire. The time span included was also discovered to be variable although participants were specifically asked for roles that were new in the last 5 years. There was also a lack of clarity about numbers of staff in particular roles in several instances, with terms such as ‘many’, ‘a few’, ‘some’, ‘lots’ used to describe data. These terms were subjective and relative, being partly dependent on the size of a Trust and partly on the interpretation of the respondent.

#### Inconsistency of Nomenclature

Many roles, often statutory ones, use consistent job titles across Trusts and in databases. However, titles for unregulated roles are more variable and may be adapted to reflect other job titles in a Trust/team. As a result, it was found that Trusts might give different names to the same role, for example, ‘Peer support workers’ and ‘Lived experience practitioners’. Some Trusts considered these titles to signify different roles, some used the terms interchangeably. The terms ‘Associate’ and ‘Assistant’ were also noted as potentially problematic by a participant in one focus group:

whether they call them associate or assistant, we’re hearing this, that it’s used, both terms are used as well (SRG 2-A).

In other instances, the same name was given to several different roles, including some which were not new, for example:

[in one Trust] all community staff were called MH practitioners. . .So it’s kind of a meaningless title. . . it’s not a new role, it’s just there is a new name of roles. (SRG2-F)

Another example of this would also be ‘Specialist Nurse Practitioner’ where the numbers provided ranged from 1 to 747 (median 82), suggesting some Trusts might have been using this as a catch-all title. It should be noted that the lack of clarity was not helped by job profiles at national level changing often (SRG2-A). For example, 1 respondent speculated:

we don't have Experience and Engagement Liaison officers but it sounds similar to the new Patient and Public Involvement Leads (Interview B1).

All of this made it difficult to compare numbers in Trusts and might also cause confusion for staff moving between Trusts.

#### Coding and Retrieval Issues with the ESR

Several interviewees in Stage 1 noted a mismatch between new roles and codes in the ESR, and a need for standardisation (C1, P1, S1). Whilst there has been a lot of progress in the last 2 years with the introduction of codes for both employed and trainee psychological practitioners, for example, several interviewees felt that the ESR still lags behind new developments (C1):

Yeah . . .we really struggle to code our psychological professions because there isn’t an actual code, so some of them get put in healthcare science, some of them get (. . .) put all over the place, which makes it really difficult to collect the data. (SRG2-B)

Examples of new roles without a code in the version of the Occupational Code Manual^20^ used at the time of the research would be Children and Young People Senior Navigator or EMHP. Coding is complex with new codes being opened and old ones retired continually, some room allowed for ‘local discretion’ (eg, with temporary changes of circumstance) and exceptions to general rules (eg, with posts that are filled by postholders from several different backgrounds). Coding is not always accurate (J1, S1, Z1) or complete (J1, P1):

Discussed numbers with the analytics team – data on ESR system is not accurate, poor quality. . .there are more people in some roles than are showing up on the system. . . For example, trainee NAs are often coded as band 3 Healthcare Assistants (HCAs), which is what they were prior to starting training. Wards use the funding from the HCA post to cover the NA post, but it’s not necessarily recorded in this way. (J1 interview summary).

SRG3-D also noted a possible difficulty when searching by name rather than a specific code:

And obviously you’ve got ESR coding now for [ACPs] but only ACPs so that’s how we’ve pulled our data. . .if we had gone into ESR and pulled data for everybody that had got ‘advanced’ in their job title it would have been much higher but probably, as a Trust, didn’t reflect what we would class as advanced clinical practitioners.

Variable use of a modifier like ‘advanced’ or substitution of a similar term could make correct retrieval problematic:

Interviewee did not have [data about CAPs] and looked through the ESR asking which would be the CAPs. We ended up looking at the numbers of “Applied Psychologist - Clinician (in training)”, the “Assistant Psychologist” and the “Assistant Clinical Psychologist” but were not clear. (P1)

SRG3-A reflected overall that they hoped a suite of new occupational codes would be an outcome of this research.

#### Inaccuracies Subsequently Found

Outsourcing of specific new roles, for example, to the third sector may also have impacted on numbers reported. This was particularly important with Peer Support Workers which were sometimes contracted out. The Trusts gave various responses for this, for example, separating the external and internal figures, or providing 1 joint figure with no further clarification. The lack of clarity indicates some uncertainty around employment status.

## Discussion

Reliable and consistent workforce data is essential if we are to understand workforce challenges, and how innovation and the strategic introduction of new roles can address these challenges.^11,13^ Several authors have noted problems with mental health NHS data including gaps in coverage, poor quality and duplicate data entry.^2,3,8,9^ Despite recent improvements such as ESR development, this paper has shown that NHS MH service workforce data on new roles suffers from many issues, a surprising number of which were identified by Dickinson et al^14^ nearly 20 years ago in their study of the implementation of new roles in mental health services:

Of particular interest are problems that relate to the criteria used to define roles, clarity over the use of terms and respondents’ apparent lack of knowledge of the whereabouts of these new staff members. ^14^ (p.10)

To the best of our knowledge this currently remains the only study specifically covering workforce data for English NHS MH new roles. Like Dickinson et al., we also identified fragmented responsibility for the data, and difficulty locating contacts who could provide the range of information required. Ambiguity of terms used, and lack of consistent nomenclature remained a problem as in 2008. Additionally coding and retrieval issues with the ESR were also noted. This made the process of obtaining the data unexpectedly difficult and time consuming, and despite considerable effort meant that some of the data retrieved was flawed and/or incomplete.

It is at present extremely difficult to obtain accurate, complete and reliable data about new roles in NHS MH services. The current situation could be improved by implementation of the following recommendations.

*NHS England*

1. There is a wide range of names used to define new roles in MH Trusts in England, and this would benefit from greater standardisation. However, it is also important to allow for some local variation in roles to meet evidenced need. Trusts could be encouraged to name locally specific roles in the context of established national role names and to a nationally-agreed specific format.

*Mental health service providers*

2. New ESR codes: progress is being made in relation to certain roles (eg, psychological practitioners), but there appears still to be a time-lag in updating the ESR. Several of these roles were identified by Health Education England as long ago as 2020.^21^ It is recommended that providers address this as soon as possible.
3. It would be helpful if a role or function was allocated in each MH Trust with responsibility for maintaining a list of all new staff roles until the ESR is able to reflect this better. Where a specific role with responsibility for new roles had been established it appeared to lead to better data being available. Without a single point of information, it is difficult for Trusts to keep oversight of continually changing complex workforce innovation.

*Researchers*

4. It is important for health service researchers to be mindful of the potential difficulties described here and to check for similar problems when collecting their research data. If they experience these problems, it would be helpful to acknowledge this and assist other researchers by raising awareness including within publications and conferences. Equally if they become aware of examples of good practice, they could also share these.

## Study Limitations

A study limitation is that we were not able to obtain data from all the NHS MH Trusts. Non-responding trusts may have experienced the same or different challenges in supplying new role data. However, the proportion of trusts supplying data (61%) was reasonable. Another limitation was the lack of definition of the term ‘new’ in the questionnaire.

## Conclusion

High quality workforce data is essential for appropriate and effective workforce planning that ensures services that meet the needs of service users. Many of the problems identified in this study have been occurring for decades. Action needs to be taken by NHS England, Trust service providers and researchers to improve the situation.

## Supplemental Material

sj-docx-1-inq-10.1177_00469580251399386 – Supplemental material for The Challenges of Searching for Workforce Data About New Roles in NHS Mental Health Services: A Cross-Sectional Observation Study Supplemental material, sj-docx-1-inq-10.1177_00469580251399386 for The Challenges of Searching for Workforce Data About New Roles in NHS Mental Health Services: A Cross-Sectional Observation Study by Sally Sanger, Damian E. Hodgson, Emily Wood, Pauline A. Nelson and Jaqui Long in INQUIRY: The Journal of Health Care Organization, Provision, and Financing
