# Supplementary material for: The Challenges of Searching for Workforce Data About New Roles in NHS Mental Health Services: A Cross-Sectional Observation Study
Source: Inquiry. 2025 Dec 1;62:00469580251399386. doi: 10.1177/00469580251399386 (PMC12669531; doi:10.1177/00469580251399386)
Supplement: sj-docx-1-inq-10.1177_00469580251399386 – Supplemental material for The Challenges of Searching for Workforce Data About New Roles in NHS Mental Health Services: A Cross-Sectional Observation Study [file sj-docx-1-inq-10.1177_00469580251399386.docx]

**Appendix A: Interview materials**

**A.1: Invitation for interviewees**

Dear

We are writing to invite you to participate in a national research project studying the impact of new roles in NHS mental health Trusts, funded by the National Institute of Health Research [ref number removed for anonymity].

The aim of the project is to learn how new roles in mental health services should be introduced, to make sure they work well for everyone. The first stage is to find out which new roles are being introduced, where and why.

To do this, we want to speak to a key person working in each mental health Trust in England who has knowledge of the new roles which have been implemented in the past few years. We anticipate that this is likely to be the head of HR, people management or an equivalent position in the Trust, and you have been approached because you have been identified as being in such a role.

Taking part in the research will involve participating in a single online or telephone interview with a researcher from the [name of University]. You will be asked to give your consent to participate in the research either prior to or at the start of the interview.

In the interview, we will ask you about the new roles that have been implemented in your Trust in the last five years, and in which teams or services new roles have been introduced. We would also like to ask about the rationale for introduction of new roles, the intended impact, and how they have been implemented. We will share with you the key questions in advance of the interview so that you will have time to reflect or check information. The interview should last about 30 minutes and will be digitally recorded. You will not be asked for any data relating to individual staff.

If you are unable to take part in an interview, we would like to send you a questionnaire to enable us to collect some basic information about the new roles introduced in your Trust. If you do not think you are the most appropriate person to take part in the research, we would be grateful if you could suggest someone else within your Trust that we could speak to.

An information sheet with further information is available by clicking the link xxx.

If you are happy to participate, we would ask you to complete the consent form by clicking the link xxx.

If you have any questions about the research, please do not hesitate to contact the principal investigators.

Yours sincerely

**A.2: Consent Form: Interviews**

| *Please tick the appropriate boxes* | Yes | No |
| --- | --- | --- |
| Taking Part in the Project |  |  |
| I have read and understood the project information sheet dated 06/07/2023 or the project has been fully explained to me. (If you will answer No to this question please do not proceed with this consent form until you are fully aware of what your participation in the project will mean.) | \|  \| \| --- \| | \|  \| \| --- \| |
| I have been given the opportunity to ask questions about the project. | \|  \|  \| \| --- \| --- \| | \|  \| \| --- \| |
| I agree to take part in the project and I understand that taking part in the project will involve taking part in an interview and/or completing a questionnaire, and that the interview will be digitally recorded. | \|  \| \| --- \| |  |
| I agree to anonymised data from the interview being used in the research. | \|  \| \| --- \| |  |
| I understand that my taking part is voluntary and that I can withdraw from the study at any point, but that after two weeks my data will be retained as it will have been anonymised and integrated into the dataset; I do not have to give any reasons for why I no longer want to take part and there will be no adverse consequences if I choose to withdraw. | \|  \| \| --- \| \|  \| |  |
| I understand that choosing to participate as a volunteer in this research does not create a legally binding agreement nor is it intended to create an employment relationship with the [name of University]. | \|  \| \| --- \| |  |
| How my information will be used during and after the project |  |  |
| I understand my personal details such as name, phone number, address and email address etc. will not be revealed to people outside the project. | \|  \| \| --- \| |  |
| I understand and agree that my words may be quoted in publications, reports, web pages, and other research outputs, but that I will not be named in these outputs. | \|  \| \| --- \| |  |
| I understand and agree that other authorised researchers will have access to this data only if they agree to preserve the confidentiality of the information as requested in this form. | \|  \| \| --- \| |  |
| I understand and agree that other authorised researchers may use my data in publications, reports, web pages, and other research outputs, only if they agree to preserve the confidentiality of the information as requested in this form. | \|  \| \| --- \| |  |
| I give permission for the specific data regarding new roles in the Trust that I provide to be deposited in the [name of repository] so it can be used for future research and learning | \|  \| \| --- \| |  |
| So that the information you provide can be used legally by the researchers |  |  |
| I agree to assign the copyright I hold in any materials generated as part of this project to [name of University]. | \|  \| \| --- \| | \|  \| \| --- \| |

| Name of participant [printed]  Name of Trust | Signature | Date |
| --- | --- | --- |

Project contact details for further information:

In the event of a complaint or concern, please contact the Head of Department.

**A.3: Participant Information Sheet: Interviews**

You are being invited to take part in a research project. This information sheet will describe exactly what this will involve and help you decide if participation is right for you and your Trust. Please read it carefully and ask us if there is anything that is not clear or you would like more information. Take time to decide whether or not you wish to take part. You may wish to discuss it with colleagues or suggest a colleague participates either as well as or instead of you. Thank you.

1. What is the project’s purpose?

New roles are being created in NHS mental health Trusts to help cope with the current staffing crisis in the service and to improve the care given to service users. However, the introduction of new roles can be challenging for everyone involved: new and existing staff, and the service users who are being supported. This project aims to discover how new roles should be introduced to make sure they work well for everyone.

The first stage of the project is to find out which new roles are being introduced, where and why *(this is the stage we are asking you to take part in)*. We will then use this information to develop a model to understand the different types of roles and why they are used and test this model with stakeholders. We will then study what happens in practice by placing researchers into mental health teams that have recently introduced new roles and speaking to staff and service users about their experience. Finally, we will use all this information to develop guidance for those managing and planning new roles in mental health services in the future. The project will last from April 2023 to the end of May 2025.

2. Why have I been chosen?

We want to interview a key person working in each mental health Trust in England who has knowledge of the new roles which have been implemented in your Trust in the past few years. This is likely to be the head of HR or similar in the Trust, and you have been approached because you have been identified as being in this role.

3. Do I have to take part?

It is up to you to decide whether you want to take part. If you decide to, you will be asked to complete a consent form to say that you are happy to take part. You can withdraw from this research without any negative consequences at any point, but after two weeks your data will be retained as it will have been anonymised and integrated into the dataset. You do not have to give a reason. If you wish to withdraw from the research, please contact [name, email, phone number] or [name, email, phone number).

*Please note that choosing to participate in this research, will not create a legally binding agreement, nor is it intended to create an employment relationship between you and [name of University].*

4. What will happen to me if I take part? What do I have to do?

Taking part in the research will involve participating in a single online or telephone interview with a researcher from the [name of University]. You can choose which method you prefer. You will be asked to give your consent to participate in the research either prior to or at the start of the interview. We will ask you about the new roles that have been implemented in your Trust in the last five years, in which teams, the rationale for their introduction, the intended impact, and how they have been implemented. You will not be asked for any data relating to individual staff. The interview should last no longer than an hour and will be digitally recorded.

If you are unable to participate in an interview, we may contact you to ask you to complete a short questionnaire covering basic information on new roles in your Trust.

*If you are unable to participate in the research, or you feel you are not the correct person to help with these questions, we would appreciate it if you could nominate a colleague who may be able to help.*

5. Will I be recorded, and how will the recording be used?

Interviews will be digitally recorded in either audio or video format. If a video recording is made, this will be converted to an audio file and the video deleted. The recording will only be used for analysis, and no-one outside the project will have access to it.

6. What are the possible disadvantages and risks of taking part?

We do not anticipate any disadvantages from taking part, except the potential inconvenience of the time taken to take part in the interview or complete the questionnaire.

7. What are the possible benefits of taking part?

While there are no direct benefits to participating in this project, the information you provide will help us conduct the later stages of the research. It is hoped that this research will generate new knowledge and practical guidance for implementing new roles in mental health Trusts which will benefit service users, staff, managers, and the organisations as a whole, as well as policy-makers

8. Will my taking part in this project be kept confidential?

All the information that we collect about you during the research will be kept strictly confidential and will only be accessible to the research team. You will not be able to be identified in any reports or publications.

9. What is the legal basis for processing my personal data?

According to data protection legislation, we are required to inform you that the legal basis we are applying in order to process your personal data is that *‘processing is necessary for the performance of a task carried out in the public interest’* (Article 6(1)(e)). Further information can be found in the University’s Privacy Notice [email address]

10. What will happen to the data collected, and the results of the research project?

The data collected for this project will only be accessible to the research team. All data will be stored in an access-restricted folder on the University's networked filestore (X: drive). Audio/video recordings will be destroyed as soon as the project is closed. All other information will be kept for 5 years in keeping with the University’s audit processes. All data used for analysis and dissemination will be fully anonymised to ensure you are not identifiable in any publications, presentations etc.

*Due to the nature of this research it is very likely that other researchers may find the data collected to be useful in answering future research questions. We will ask for your explicit consent for your data to be shared in this way*. If you agree to us sharing the information you provide with other researchers (e.g. by making it available in a data archive) then your personal details will not be included.

11. Who is organising and funding the research?

[Data redacted for anonymity]

12. Who is the Data Controller?

The [name of University] will act as the Data Controller for this study. This means that the University is responsible for looking after your information and using it properly.

13. Who has ethically reviewed the project?

This project has been ethically approved via the [name of University]’s Ethics Review Procedure, as administered by [name of School]. It has also received HRA approval, [redacted for anonymity].

14. What if something goes wrong and I wish to complain about the research or report a concern or incident?

It you have any concerns about this research or wish to make a complaint, please contact [names], who are responsible for this study. If you feel your complaint has not been handled satisfactorily, you can contact the Head of Department, [name and email], who can escalate the complaint through the appropriate channels.

If the complaint relates to how your personal data has been handled, you can find information about how to raise a complaint in the University’s Privacy Notice:[web address]

If you have any safeguarding concerns, please contact [name] the Designated Safeguarding Contact for the research.

15. Contact for further information

For more information about the project, please contact either of the following:

[names, email addresses and phone numbers]

Thank you for agreeing to participate in our research. We are grateful for your time and valuable contribution.

**A.4: Interview topic guide**

**Introduction**

- Remind re purpose of interview, answer question
- Do consent process if not already done
- Confirm agreement to record, not transcribed.
- Confirm all info will be used anonymously
- Ask for name and role, how long been in role

**Main topic areas** (additional follow-up questions will be added as needed to explore the details )

1. Which new roles have been introduced into your Trust over the last 5 years?
    List all known ones: names, number in role, Agenda for Change banding.
2. Describe the nature of the roles e.g. entirely new role or change to existing role, how long have they been established, etc?
3. In which teams or services/settings have these been introduced?
4. What was the rationale for introducing these roles? Strategic/policy aim?
5. What was the intended impact of the roles? On staff, service users?
6. What approaches have been taken to embedding and supporting these roles? How were decisions made about implementation? How effective have these approaches been? Has any evaluation been undertaken?
7. Do you think there may be other new roles that have been introduced that you are unaware of? Is there anyone else you think we need to approach/ask for information?

Completion

- Thanks
- Reminder re confidentiality
- Reminder re withdrawing, use of data

**Appendix B**

**National Mapping of Patient-Facing New Roles in Mental Health Trusts: Questionnaire**

1. Name of Trust (for administration purposes only):
2. Name and role of individual completing the form (for administration purposes only):
3. Which of the following new roles are currently employed in your Trust? For each role, please indicate whether or not you employ staff in this role, and as far as possible please indicate the number of staff employed and the Agenda for Change banding if known.

| **Role** | **Y/N** | **Number in role** | **AfC banding** | **Role** | **Y/N** | **Number in role** | **AfC banding** |
| --- | --- | --- | --- | --- | --- | --- | --- |
| Advanced Practitioner |  |  |  | Experience & Engagement Liaison Officer |  |  |  |
| Clinical Associate Psychologist |  |  |  | Family Ambassador |  |  |  |
| Physician Associate |  |  |  | Youth Intensive Psychological Practitioner |  |  |  |
| Nursing Associate |  |  |  | Children & Young People Senior Navigator |  |  |  |
| Peer Support Worker |  |  |  | Children & Young People Keyworker |  |  |  |
| Specialist Nurse Practitioner |  |  |  | Education MH Practitioner |  |  |  |
| Assistant Practitioner |  |  |  | Employment Specialist |  |  |  |
| Wellbeing Practitioner (or similar) |  |  |  | Employment Support Worker/Adviser |  |  |  |
| Mental Health Practitioner |  |  |  | Creative Practitioner |  |  |  |
| Health & Wellbeing Triage Coach (or similar) |  |  |  | Drama Therapist |  |  |  |
| MH & Wellbeing Navigator (or similar) |  |  |  | Art Therapist |  |  |  |
| Community Connector |  |  |  | Music Therapist |  |  |  |
| Lived Experience Worker |  |  |  | Telephone Triage Worker |  |  |  |

1. Please provide details of any other new roles that have been introduced in your Trust that are not included above.

| **Role** | **Number in role** | **AfC banding** |
| --- | --- | --- |
|  |  |  |
|  |  |  |
|  |  |  |
|  |  |  |
|  |  |  |
|  |  |  |
|  |  |  |
|  |  |  |
|  |  |  |
|  |  |  |

1. Please add any further comments or clarification relating to job titles and banding of new roles. We would also be grateful for any information about how long the roles have been established and in which teams/services

|  |
| --- |

1. We would be interested in any other information about your experience of introducing new roles in your Trust. For example, what was the rationale for their introduction? How were decisions made about implementation? What approaches have been taken to introducing and embedding them? What challenges or facilitators have you identified? Has there been any evaluation?

|  |
| --- |

Thank you for taking the time to complete this questionnaire. Your help is much appreciated. If you have any questions please contact [email address]
